# DIGITAL NARRATIVE GERONTOLOGY AS BRIDGES BETWEEN GENERATIONS TO IMPROVE WELL-BEING, LEARNING, AND SHARED EXPERIENCES

**DOI:** 10.1093/geroni/igad104.0789

**Published:** 2023-12-21

**Authors:** Béatrice Crettenand Pecorini, Emmanuel Duplàa

**Affiliations:** University of Ottawa, Ottawa, Ontario, Canada; University of Ottawa, Ottawa, Ontario, Canada

## Abstract

Elders are an essential society component for the collective success of the demographic transition, for social inclusion and the fight against ageism (Berrut, 2020). Ageism - discrimination by age, is a very present concern. One way to respond to this challenge is to build bridges between generations in society; intergenerational programs can reduce the perception of stereotypes about other generations (Barbosa et al., 2021; Pentecouteau & Eneau, 2017), strengthen intergenerational solidarity, and help develop capital and social cohesion (Topping, 2020). Based on the theories of lifelong learning and intergenerational learning, our research engages digital narrative gerontology (Crettenand Pecorini, 2019). We have organized several meetings in pairs (elder – young) where each pair created a digital narrative from the oral narration of the elder life story, supported by the multimedia skills that the young adult acquired during the workshop we have set up. From individual and pairs semi-directed interviews, logbooks, debriefings, and digital narrative, based on case study thematic analysis, our preliminary results confirm the results obtained in 2019: improved general well-being, reduction in the perception of generation stereotypes, reduction in feelings of loneliness and isolation, strengthening intergenerational solidarity, satisfaction to rediscover one’s life or the projection of one’s life stimulated by example, new knowledge acquisition, as well as the pride of artifact creation and its sharing. This innovative project can be applied in schools, colleges, and universities as well as in community centers and seniors’ residences.

